# Proteome-wide mendelian randomization reveals circulating proteins causally associated with childhood body mass index

**DOI:** 10.1038/s41598-025-31836-y

**Published:** 2025-12-15

**Authors:** Raphael Avocegamou, Basile Jumentier, Kaossarath Fagbemi, Nahid Yazdanpanah, Mojgan Yazdanpanah, Isabel Gamache, Despoina Manousaki

**Affiliations:** 1 Azrieli Research Center of the Sainte-Justine University Hospital, CHU Sainte Justine, Université de Montréal, 3175 Côte-Sainte-Catherine, Montreal, Quebec H3T 1C5 Canada; 2 Department of Biochemistry and Molecular Medicine, Université de Montréal, CHU Sainte Justine, 3175 Côte-Sainte-Catherine, Montreal, Quebec H3T 1C5 Canada; 3 Department of Pediatrics, Université de Montréal, CHU Sainte Justine, 3175 Côte-Sainte-Catherine, Montreal, Quebec H3T 1C5 Canada

**Keywords:** Proteins, Pediatric obesity, Mendelian randomization, Causal inference, Biomarkers, Computational biology and bioinformatics, Diseases, Genetics, Medical research

## Abstract

Childhood obesity is a major public health problem, affecting one in 5 youths. We aimed to characterize biomarkers for pediatric obesity among circulating proteins using Mendelian randomization (MR). We utilized genome-wide significant cis-protein quantitative trait loci (pQTL) from three large adult proteomic GWAS (N total>58,000) and a small childhood proteomic GWAS (N=2,147) as genetic instruments for circulating protein levels. Using two-sample Mendelian randomization, we estimated causal effects of the circulating proteins on childhood body mass index (BMI) in a European GWAS of 39,620 children. MR Wald ratios were calculated to estimate the causal effect of each protein on childhood BMI. Sensitivity analyses testing the MR assumptions included colocalization and phenome-wide association studies (PheWAS). Replication was conducted using independent GWAS datasets, complemented by reverse MR and tissue enrichment analyses. Among 535 tested proteins, three colocalized and demonstrated decreasing effects on BMI per standard deviation increase in their level: endoglin (ENG; MR beta: -0.07, 95% CI [-0.10, -0.04], P=4.4×10⁻^5^), fatty acid binding protein 4 (FABP4; MR beta: -0.33, 95% CI [-0.50, -0.16], P=1.3×10⁻^4^), and cell adhesion molecule 1 (CADMI1; MR beta: -0.26, 95% CI [-0.37, -0.15], P=5.45×10⁻^5^). All three proteins showed evidence of colocalization (posterior probability >75%) and were identified using adult proteomic GWAS, given a limited statistical power using the pediatric proteomic GWAS data. Reverse causation was identified for FABP4, suggesting a compensatory mechanism. In conclusion, we identified three circulating proteins as potential blood biomarkers or drug targets for pediatric obesity, warranting further functional validation to elucidate biological mechanisms and assess therapeutic potential.

**Supplementary Information:**

The online version contains supplementary material available at 10.1038/s41598-025-31836-y.

## Introduction

Childhood obesity is a global health issue^1^ affecting almost one in five children and has been acknowledged as a serious public health concern due to its high morbidity rates^2^. In comparison to children with a normal weight, children with obesity, defined as having a body mass index (BMI) two standard deviations above the age and sex-adjusted mean, have an increased likelihood of living with obesity in adulthood^3^ and are at a higher risk of developing long-term cardiometabolic, psychosocial and musculoskeletal complications^4,5^. While there is a partial genetic overlap between adult-onset and pediatric-onset adiposity, childhood obesity appears to have distinct genetic determinants and eventually predictive biomarkers^6^. The identification of early biomarkers specific for childhood-onset obesity is key for the development of screening tools or new therapies and for the repurposing of existing drugs.

With the advance of high throughput proteomics, circulating proteins represent a valuable source for biomarker discovery, because their circulating abundances are measurable and possibly modifiable. For instance, the Glucagon-like peptide-1 (GLP-1) is a peptide detected in both the intestines and the blood that has been an approved therapeutic target for both adult and pediatric obesity^7^. However, measuring serum proteins is limited by prohibitive costs, measurement errors, bias due to unmeasured confounding and reverse causation^8^, and importantly for our study, the small sample sizes of available pediatric cohorts. Nevertheless, a recent pediatric study studying the genetic determinants of ~1200 protein levels in over 2000 children of European ancestry showed excellent replication in 558 adults, which implies that the genetic effects on plasma protein levels persist from childhood into adulthood^9^. These findings support the validity of using adult-derived pQTLs as instruments for pediatric outcomes in Mendelian Randomization studies.

Mendelian randomization (MR) is a method providing an instrumental variable framework to mitigate the above biases and infer causality^10,11^. This approach utilizes genetic variants, randomly allocated at conception, as instruments for a biomarker to evaluate the causal impact of this biomarker on a disease or a trait. MR leans on three pivotal assumptions^10,11^. First, the genetic instrument must be robustly associated with the exposure, known as the relevance assumption. Second, the genetic instrument should lack association with confounding factors influencing the relationship between the exposure and the outcome, known as the independence assumption. Third, the genetic instrument should not influence the outcome through alternative pathways unrelated to the exposure, termed the exclusion restriction assumption. The violation of the last assumption is known as horizontal pleiotropy.

Recent expansive genome-wide association studies (GWAS) in adults have identified optimal genetic instruments for circulating protein levels within the gene encoding the protein, termed *cis*-protein quantitative trait loci (*cis*-pQTL)^12–14^. Their proximity to genes encoding proteins make the *cis-*pQTL ideal MR instruments, by minimizing the possibility of horizontal pleiotropy. Thus, prior MR studies have explored causal relationships between circulating protein levels and a variety of complex diseases and traits mostly in adults^15–18^.

In this study, we undertook an integrative proteogenomic analysis to systematically identify potential biomarkers for pediatric BMI. First, using *cis*-pQTL for circulating proteins from adult and pediatric GWAS, we estimated the causal effect of genetically altered levels of these proteins on pediatric BMI among 39,620 children from a large European GWAS^19^. We then prioritized the candidate proteins through sensitivity analyses addressing potential horizontal pleiotropy and testing Bayesian colocalization and replication in independent cohorts. Furthermore, we identified target tissues through enrichment analyses.

## Results

### Main Mendelian randomization analyses

**Fig. 1:**
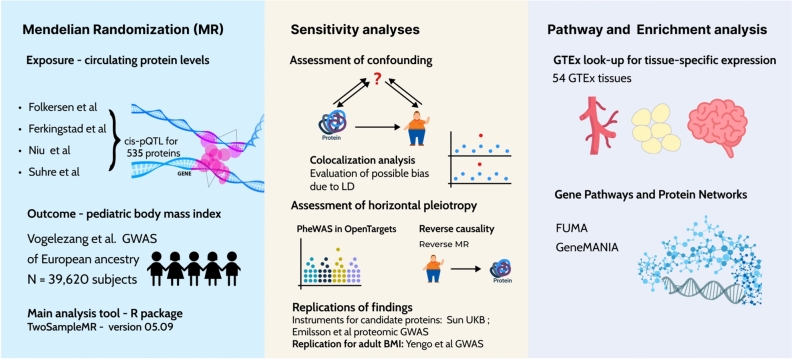
Overview of the study. Mendelian randomization (MR) was conducted using the Wald ratio to estimate the effect of each circulating protein on pediatric BMI. Colocalization analyses and evaluation of horizontal pleiotropy through annotation and phenome-wide association study (PheWAS) were conducted to verify the MR assumptions. Significant associations were replicated using other proteomic studies and an adult BMI GWAS. Gene expression enrichment analyses were conducted to identify potential target tissues and cell types.

**Fig. 2:**
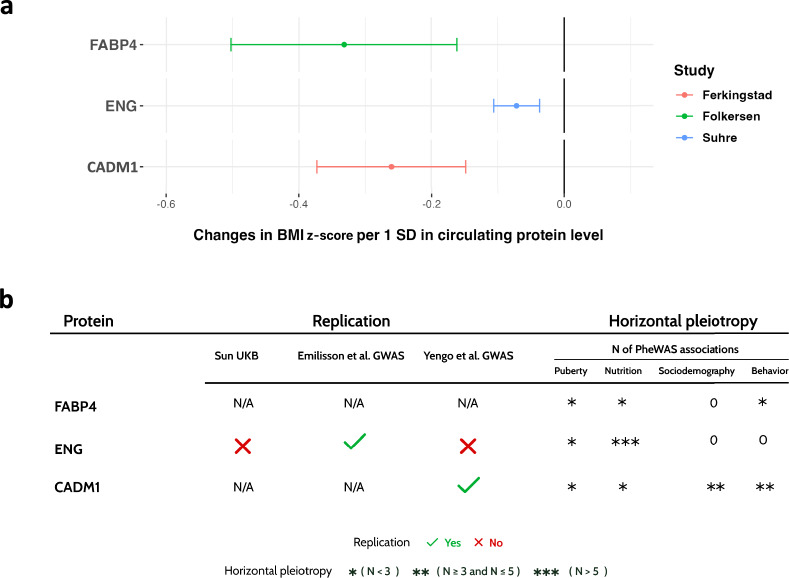
Summary of the study’s results. a. Forest plot illustrating the MR associations between the three candidate proteins and pediatric BMI. The *β* coefficients indicate the impact of a 1 SD increase in the protein level, as determined by its genetic instrument, on standardized BMI. The color code indicates the GWAS source of the genetic instrument. b. Candidate protein prioritization. Associations supported by strong or suggestive colocalization evidence were replicated using additional GWAS (Replication panel). The N of associations with confounding traits of the genetic instrument of each protein in the PheWAS analyses is depicted in the left panel (Horizontal Pleiotropy panel).

We performed two-sample Mendelian randomization (MR) to estimate the causal effect of genetically predicted circulating protein levels on childhood BMI. (Fig. 1). First, following identification of *cis*-pQTL instruments for 2,130 proteins in two adult and one childhood protein GWAS^9,19^ (Supplementary Table 1), we performed data harmonization (Supplementary Table 2), which enabled MRs for 535 circulating proteins. All *cis*-pQTL had an F-statistic >10. Results of these MR analyses are provided in Supplementary Table 2. Three MR associations reached an FDR-corrected significant p-value below 0.05 (Fig 2a). These are the endoglin (ENG; MR beta: −0.07, 95% CI= [−0.10, −0.04], P= 4.4 x10^−5^), the fatty acid binding protein 4 (FABP4; MR beta: −0.33, 95% CI= [−0.5, −0.16], P=1.3 x10^−4^), and the cell adhesion molecule 1 (CADM1; MR beta: −0.26, 95% CI= [−0.37, −0.15], P=5.45 x10^−5^), all demonstrating a negative effect on standardized BMI per one standard deviation increase in their blood level. No significant MR associations were found using *cis*-pQTL for 63 proteins (out of the total 535 proteins) from the only available pediatric proteomic GWAS^9^ (Supplementary Table 2). The statistical power of the MR analyses are displayed in Supplementary Table 3. Of note, our MR analysis using instruments from the sole childhood proteomic GWAS indicated a power of only 13% to detect the largest beta coefficient obtained in our MR results using adult protein GWAS.

### Sensitivity analyses testing the MR assumptions (confounding and horizontal pleiotropy assessment)

**Fig. 3:**
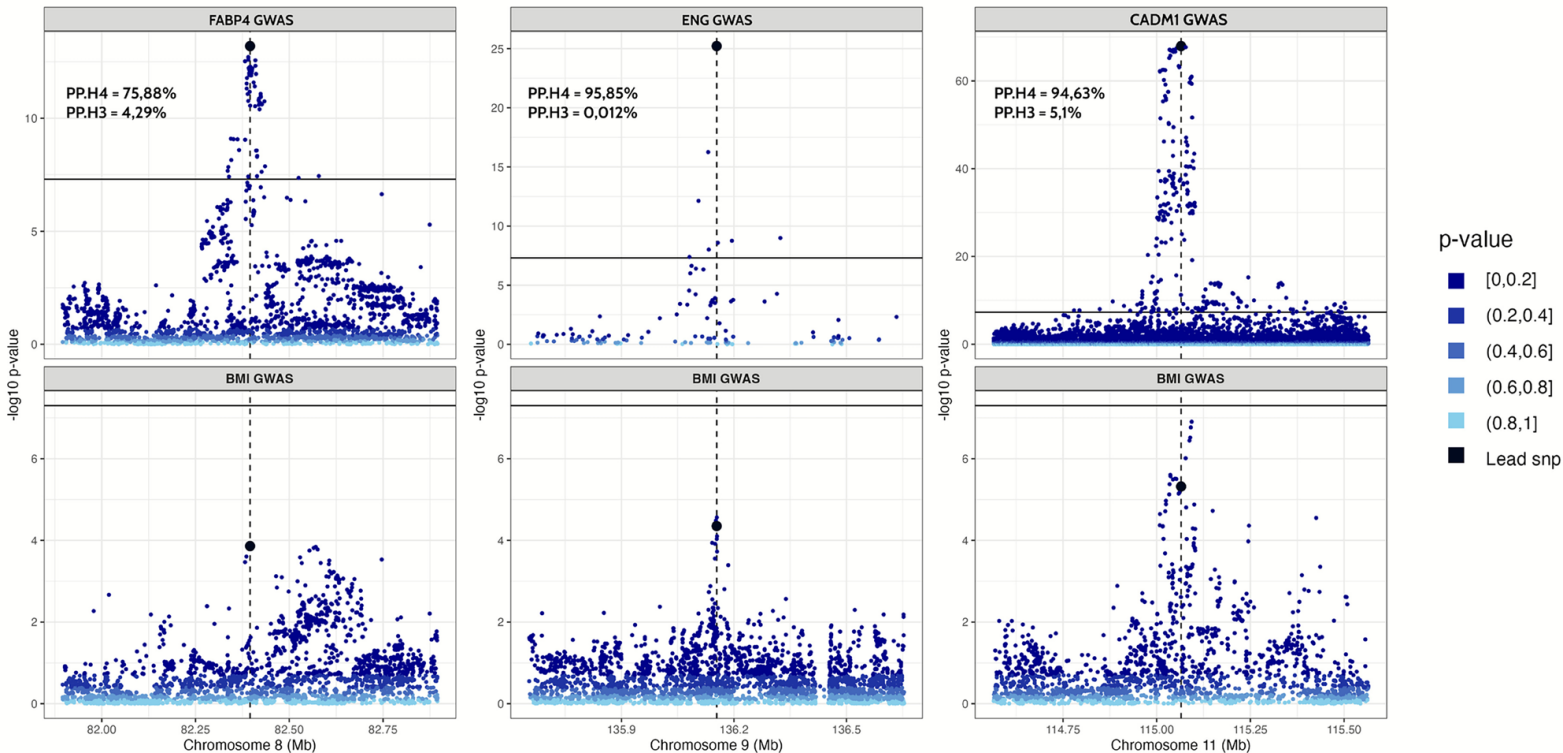
Colocalization of genetic associations with candidate proteins and the pediatric BMI. The lead cis-genetic instruments are indicated. Genetic variants located in a ±500kb window centered around each genetic instrument are plotted with their significance in respective studies with the corresponding instrument. For each target protein, the posterior probability of colocalization (PP.H4) and the posterior probability of co-existence of two distinct causal variants (PP.H3) are indicated.

To test whether protein and childhood BMI associations arose from shared causal variants rather than linkage disequilibrium, we performed Bayesian colocalization analyses using the coloc R package. Colocalization analyses for the three MR-prioritized proteins showed moderate to high evidence of colocalization with H4 exhibiting the highest posterior probabilities for ENG (*cis*-pQTL: rs651007, H4=96%), followed by CADM1 (*cis*-pQTL: rs11215406, H4=95%) and FABP4 (*cis*-pQTL: rs77878271, H4=76%) (Fig 3**, **Supplementary Table 4).

To assess pleiotropy or confounding, we carried out phenome-wide association studies (PheWAS) for the MR instruments using *OpenTargets* data (https://www.opentargets.org). We identified that the single genetic instruments of ENG, FABP4, and CADM1 have been associated in GWAS with various traits linked to childhood obesity, including anthropometric, pubertal, sociodemographic, and nutritional traits (Fig 2b** and **Supplementary Tables 5-7). This may have introduced potential bias to our MR studies, due to these traits acting as confounders of the MR associations or introducing horizontal pleiotropy in the MR instruments of the three candidate proteins.

### Replication and reverse MR analyses

We conducted replication MR analyses using independent instruments from additional proteomic GWAS, including the UK Biobank Pharma Proteomics Project by Sun et al^20^ and a previous GWAS by Emilsson et al^21^, to validate the initial associations. Using *cis*-genetic instruments identified in these GWAS only the association for ENG replicated (Fig 2b and Supplementary Table 8). Additionally, by testing effects of the candidate proteins on adult BMI, using data from a GWAS by Yengo et al^22^ (Fig 1), we observed a MR effect on adult BMI for CADM1, exhibiting a consistent direction and magnitude of effect as observed in our pediatric MR analysis (beta = −0.08, p = 1.973×10^−9^), while ENG obtained a suggestive p-value (p = 0.049). We could not replicate the result for FABP4 since the *cis*-pQTL instrument for this protein (or a proxy) was not found in the adult BMI GWAS (Fig 2b and Supplementary Table 8). Of note, while two out of the three proteins (ENG and CADM1) were measured in the pediatric study^9^, no available SNP-instruments could be found in this GWAS for a replication MR.

To assess reverse causality, we performed MR using BMI-associated SNPs as instruments and tested their effects on circulating protein levels. Using 16 SNPs as instruments for BMI following clumping and data harmonization, our reverse MR analyses revealed evidence of a reverse association of BMI with FABP4 (MR beta: 0,24, 95% CI =[0.17, 0.33], P = 1.803 x 10^−9^ in the IVW method and MR beta: 0,28, 95% CI=[0.17, 0.40], P = 1.814x10^−6^ in the Weighted Median method). Conversely, the results for CADM1 in the reverse MR analyses were not statistically significant (P= 0.44). The MR-Egger intercept of these analyses confirmed the absence of horizontal pleiotropy. However, we note that we were unable to retrieve effects of the BMI SNPs in the GWAS for the ENG protein which prohibited undertaking reverse MR testing for this protein (Supplementary Table 9).

### Two-Step network mendelian randomization analysis

Given the fact that the single *cis*-pQTL instruments for each of the three candidate proteins were associated with anthropometric traits, which could induce bias due to pleiotropy to our MR associations, we explored the presence of mediation by adiposity using a two-step network MR approach. First, we estimated the causal effect of candidate proteins on body fat percentage (%BF) using data from an adult GWAS on BF^23^, then we estimated the effect of %BF on pediatric BMI. Our two-step network MR analysis indicated that the effects of the candidate proteins on BMI were not mediated by %BF, as evidenced by the non-significant p-values observed in the mediation analysis (Supplementary Table 10). Consequently, we concluded that the impact of these proteins on BMI is likely independent of their effects on adiposity.

### Pathway and enrichment analyses

**Fig. 4:**
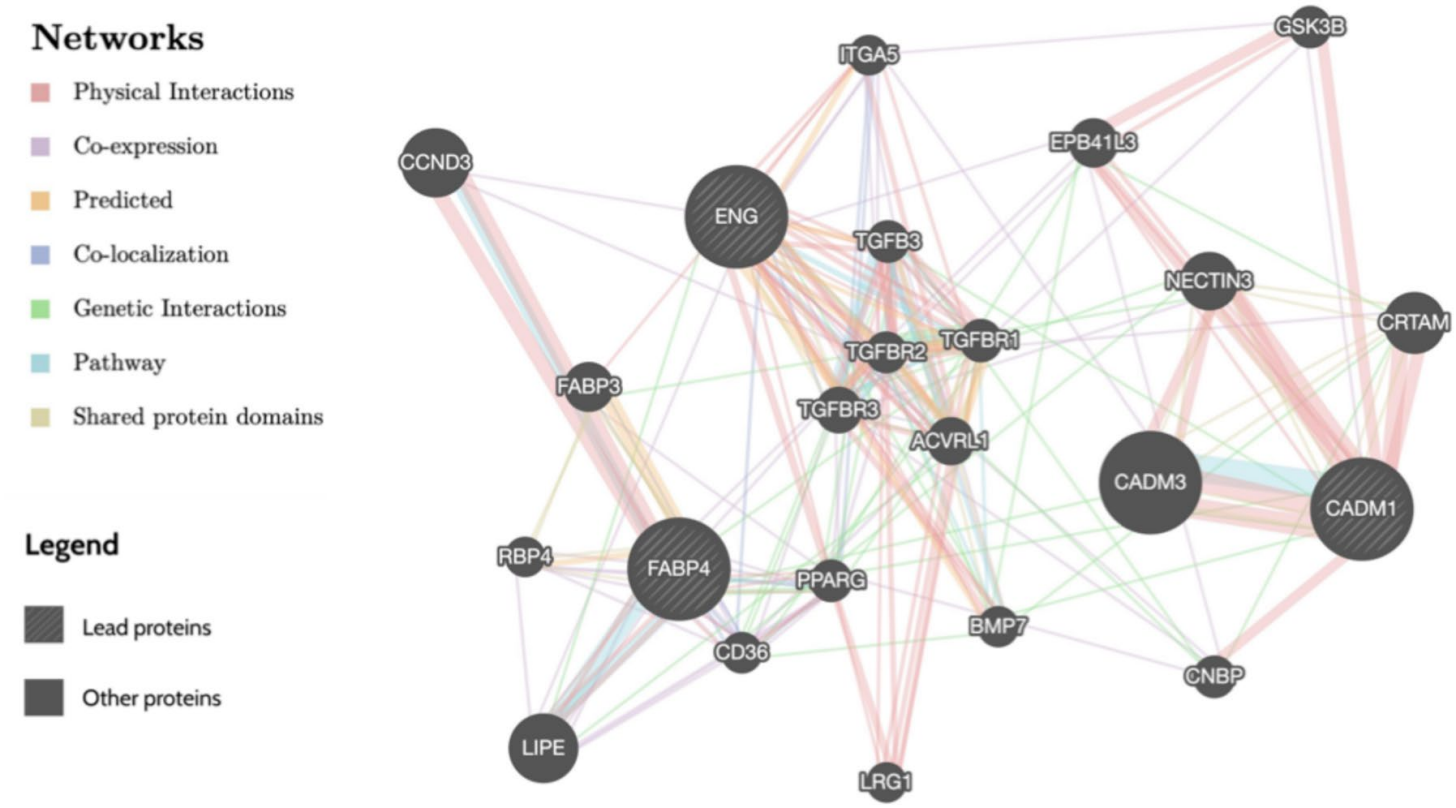
The genes of the candidate proteins are depicted as larger inner circles, while genes identified through the GeneMANIA extension are shown as smaller outer circles. Interaction types are color-coded: red lines indicate Physical interactions, purple lines denote Co-expression, green lines represent Genetic Interactions, and blue lines signify Pathway involvement. Node sizes, shown in dark gray, reflect the weights of these genes, with larger nodes indicating higher weights.

**Fig. 5:**
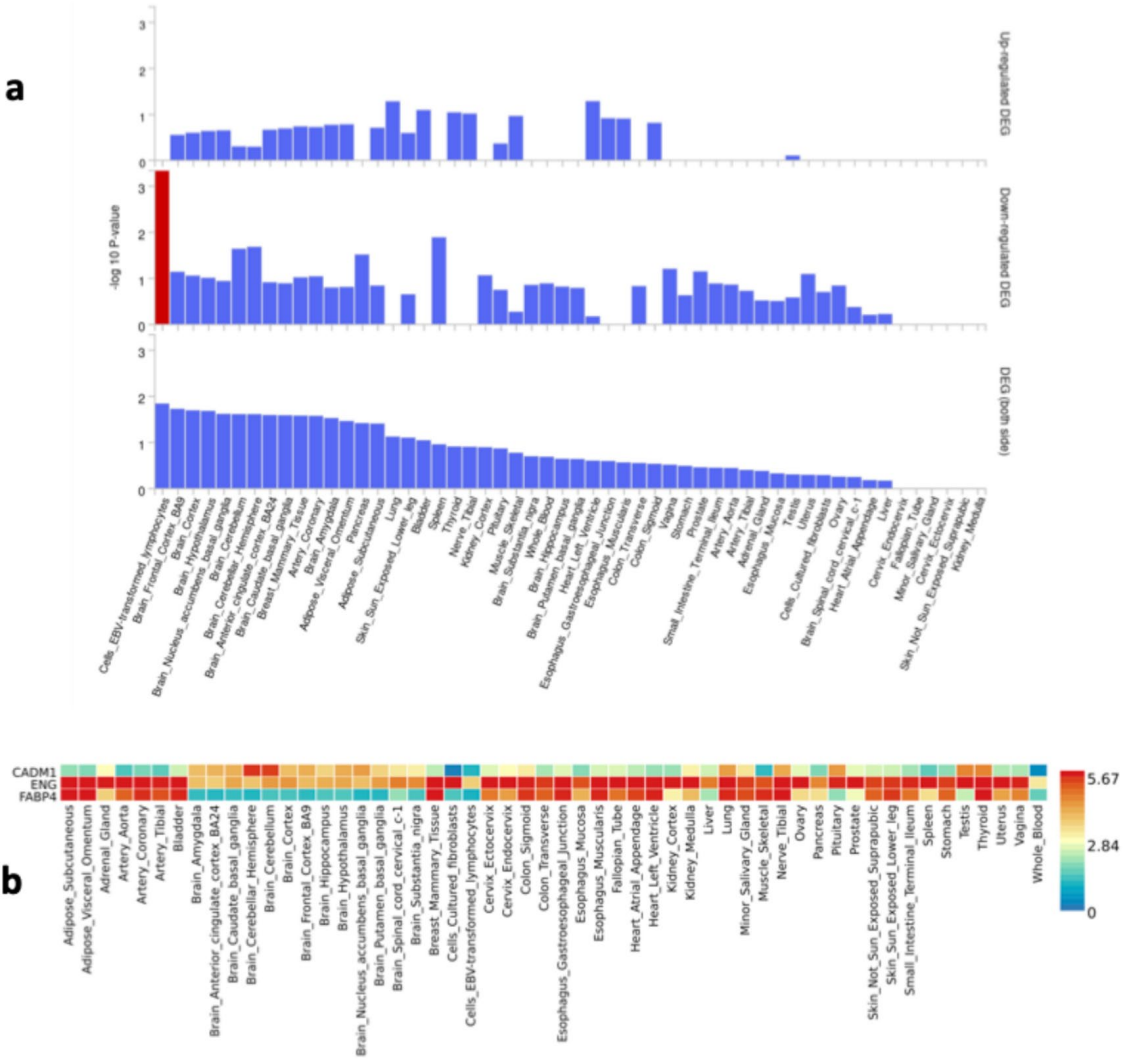
Tissue expression analyses for the genes of the three prioritized proteins. Graph a displays the normalized expression values (zero-mean normalization of log2-transformed expression), where darker red indicates higher relative gene expression within each label, and darker blue signifies lower expression. Graph b depicts the -log10 p-values for Differentially Expressed Genes (DEGs) in each dataset. 'Up-regulated’ refers to over-expression, while 'Down-regulated’ indicates under-expression.

To interpret the biological roles of the prioritized proteins, we performed pathway enrichment using GeneMANIA and Metascape, and assessed expression patterns using FUMA GTEx data. Our GeneMANIA analysis revealed that the genes associated with the three candidate proteins exhibit physical interactions, co-expression, co-localization, or shared biological pathways (Fig 4**, **Supplementary Tables 11-13). Further exploration with Metascape highlighted associations of *FABP4* and *CADM1* with cancers (head and neck), while *ENG* was linked to congenital malformations of the circulatory system. Using FUMA, we found that *ENG* is predominantly expressed in vascular walls, while *FABP4* and *CADM1* showed overexpression in the brain and adipose tissue (Fig 5**, **Supplementary Table 13).

Finally, in *OpenTargets* (Supplementary Table 13), ENG emerged as a target for an existing drug, Carotuximab. Carotuximab, also known as TRC105, is a monoclonal antibody tested in clinical settings primarily for its anti-angiogenic properties in cancer treatment, underscoring a potential drug repurposing relevance of ENG^24^.

## Discussion

Childhood obesity is an important public health problem with substantial implications for long-term health outcomes and impacts on healthcare systems worldwide. In this study, assuming that the genetic determinants of protein levels are stable throughout the life course, we sought to uncover biomarkers specific to pediatric obesity among circulating proteins using a combined approach based on Mendelian randomization and supported by colocalization and a series of sensitivity analyses. Our findings identified three circulating proteins, ENG, FABP4, and CADM1, which exhibited protective effects on childhood obesity with CADM1 also demonstrating an effect on adult BMI in the same direction. By undertaking a reverse MR analysis, we identified a probable compensatory effect of pediatric obesity on levels of FABP4. Our two-step MR highlighted that the impact of these proteins on BMI is likely not explained by direct effects on adiposity. Our PheWAS indicated possible pleiotropic bias in all three MR associations, therefore our results should be interpreted with caution. However, these findings contribute to the existing literature on the potential roles of ENG, FABP4, and CADM1 in metabolic regulation and adipose tissue function.

Among the three prioritized proteins, ENG emerged as the protein with the strongest evidence of association with pediatric BMI and is a known drug target in humans (Supplementary Table 13). Endoglin (ENG) serves as a coreceptor for transforming growth factor (TGF)-β1 and TGF-β3, and it is highly expressed on cell membranes of endothelial cells (ECs) and syncytiotrophoblasts^25^. Beyond its canonical role in angiogenesis and vascular development, ENG is expressed by various cell types, including endothelial cells, vascular smooth muscle cells, fibroblasts, hepatic stellate cells, and activated macrophages^26–29^. In our analysis, we found that ENG expression was enriched in vascular walls. While endothelial dysfunction associated with adult obesity contributes to the development of cardiovascular diseases such as hypertension, atherosclerosis, and metabolic syndrome, evidence also suggests that endothelial dysfunction can precede and potentially contribute to the development of obesity, especially in children and adolescents^30^. Indeed, our reverse MR study for ENG did not provide evidence of effects of childhood BMI on ENG levels. The above underscore the importance of further functional studies to elucidate the role of ENG in pediatric or adult obesity.

FABP4, also known as Fatty Acid Binding Protein 4 or AP2 (adipocyte protein 2), is a circulating protein showing a protective effect against pediatric obesity in our MR analysis. Interestingly, our reverse MR analysis indicates a positive causal effect of pediatric BMI on FABP4 levels, which is in contrast with the direction of effect in our main MR findings. FABP4 is primarily expressed in adipocytes and macrophages, playing a significant role in lipid metabolism and inflammatory responses^31^. Its expression is often upregulated in individuals with obesity and correlates with insulin resistance and metabolic disorders, under the action of inflammatory cytokines^32^. When fabp4 deficient mice were placed on a high-fat, high-caloric diet, the total weight gain in these mice was higher than that in wildtype controls^33^. Another study showed that pharmacological inhibition of fabp4 in mouse models can prevent atherosclerosis and type 2 diabetes^31^. In our study, we observed overexpression of FABP4 in adipose tissue, indicating its potential involvement in central and peripheral mechanisms of energy balance regulation^34^. The upregulation of FABP4 levels in obesity, as indicated by our reverse MR analysis and the existing literature^35^, suggests a possible compensatory effect (canalization) underlying the complex interplay between adipose tissue function and metabolic homeostasis.

Cellular adhesion molecule 1 (CADM1) is another circulating protein negatively associated with both pediatric and adult BMI in our MR analyses. CADM1, a cell adhesion molecule belonging to the Nectin family, is involved in various cellular processes, including cell-cell adhesion, migration, and signaling and has been implicated in tumorigenesis in humans and mice^36^. While its specific role in metabolic regulation remains elusive, CADM1 has been implicated in diverse physiological functions across different tissues and organs^37^. Interestingly, it plays a significant role in immune recognition by acting as a ligand for the immune receptor CRTAM, which is predominantly expressed on cytotoxic lymphocyte cells, implicating it in the intricate balance between cell adhesion and immune system interactions^38^. This dual functionality of CADM1 as a regulator of cellular processes and an immune regulator may indicate its broader role in metabolic homeostasis, potentially affecting pathways linked to pediatric obesity.

Our study has several strengths. A principal advantage is our combined approach based on MR and colocalization, further complemented by replication MRs in independent cohorts, sensitivity analyses for pleiotropy, and pathway and enrichment analyses. These complementary approaches enhanced our ability to uncover causal associations by integrating diverse types of data and to elucidate shared biological pathways among the candidate proteins and increase the credibility of the identified causal associations. This replication across various protein measurement platforms and study populations underscores the reliability and generalizability of our findings. Furthermore, the roles of ENG, FABP4, and CADM1 in critical biological processes such as vascular function, lipid metabolism, immunity, and adipose tissue regulation highlight the complex, and to some extent distinct etiology of childhood obesity from that of obesity presenting later in life.

Nevertheless, our study presents some important limitations. Despite leveraging large-scale GWAS datasets and robust statistical methods, the inherent constraints of MR analysis, such as potential pleiotropy and unmeasured confounding, cannot be entirely excluded^39^ especially given the results of our PheWAS analysis. The broad PheWAS associations of the genetic instruments of the three proteins suggest that the results of our causal analysis should be interpreted with caution^40^. In this direction, variations in the levels of these proteins might affect BMI through associations with pubertal timing, lifestyle habits or the built environment rather than being themselves the underlying causes of childhood obesity. The two-step MR showing absence of mediation by adiposity further implies the presence of adipose-independent pathways explaining the MR effects of these proteins on childhood obesity.

Another important limitation is that the SNP-instruments (*cis*-pQTL) utilized in the three significant MR associations were derived from adult GWAS, based on the assumption that the genetic variants influencing protein levels are stable across the life course. This is inherently contradicting the fact that in the MR framework, the exposure should precede the outcome. However, recent evidence indicates that many *cis*-pQTLs remain stable across the life course^9^, but this has not been verified for the three candidate proteins. Regarding cross-age validation of pQTL effects, we note that comprehensive multi-tissue gene expression data could support the stability of the *cis*-pQTL effects throughout the lifecourse. Unfortunately, expression data from healthy pediatric samples are not yet publicly available. The developmental GTEx (dGTEx) project aims to address this gap^41^ in the future. Despite this limitation, the Niu et al. proteomic study provides robust evidence that genetic effects on protein levels remain highly stable from childhood through adulthood, with 91−97% replication and r > 0.97 effect size concordance. For FABP4 specifically, pediatric genetic studies have confirmed that variants influence protein levels and metabolic phenotypes in children with effect directions consistent with adult findings^42^. Finally, despite a stability of genetic effects on protein levels across age groups, we acknowledge that proteome abundance and regulation vary substantially across the lifespan^43,44^. These age-related changes occur through environmental influences and post-transcriptional regulatory mechanisms that operate independently of the genetic architecture captured by *cis*-pQTLs. Future studies should investigate whether the causal effects of these proteins on childhood obesity differ in magnitude across developmental stages, as protein-disease relationships may be modified by age-specific biological contexts.

While we also used SNP-instruments from the sole pediatric proteomic GWAS in our discovery MR, the small sample size of this GWAS has precluded detection of positive associations. Our power analysis indicated approximately 13% statistical power to detect effect sizes of the magnitude we observed in our main analyses (Supplementary Table 2). This low power substantially increases the probability of Type II errors. Consequently, the absence of statistically significant associations in the pediatric proteomic replication cohort should not be interpreted as evidence against those associations, but rather reflects insufficient power to detect true effects of small to moderate magnitude. Based on our observed effect sizes, approximately 15,000–20,000 pediatric participants would be required to provide 80% statistical power for rigorous replication. As emphasized in the statistical literature^45^, absence of evidence is not evidence of absence, particularly in underpowered studies. Our findings should therefore be viewed as hypothesis-generating, requiring validation in larger pediatric proteomic cohorts as they become available.

Finally, our analysis was restricted to European populations, limiting the generalizability of our findings to other ethnic and demographic groups.

In conclusion, our study identified three circulating proteins linked to pediatric obesity using Mendelian randomization. The identification of ENG, FABP4, and CADM1 may open new avenues for research and clinical translation in the field of pediatric obesity. These findings provide genetic evidence supporting biological relevance of these proteins to childhood obesity and highlight them as promising candidates for functional validation studies. Future studies should focus on validating these findings using large pediatric protein GWAS data, and ancestrally diverse pediatric populations. Upon such validation and the elucidation of the underlying biological mechanisms of these molecules, these discoveries can inform future therapeutic interventions to mitigate the burden of pediatric obesity.

## Methods

Our study adheres to the MR-STROBE^10,11^ checklist (Supplemental Material) and did not require ethics approval.

### Study exposures (cis-pQTL GWAS)

The instrumental variables (*cis*-pQTL) for circulating proteins were derived from three proteomic GWAS in adults of European descent^14,20,21^ and one GWAS in children of European descent^9^. *Cis*-pQTL were identified as single-nucleotide polymorphisms (SNPs) that were independently associated with the protein levels (p ≤5 x 10^−8^) and located within 1 Mb of the transcription start site of the protein coding gene. To satisfy the first MR assumption, we retained *cis*-pQTL with an F-statistic >10 defining a strong MR instrument. The measurements of circulating proteins in the Ferkingstad et al.^14^ (N= 35,559), and Suhre et al.^46^ GWAS (N=1000) were conducted using the SomaLogic platform, while the Folkersen et al^47^ GWAS (N=21,758) and the Niu et al.^9^ pediatric GWAS (N=2,147) employed the Olink platform (Supplementary Table 1**)**.

### Study outcomes (BMI GWAS)

To evaluate the association between *cis*-pQTL and pediatric BMI, we retrieved their effects from a large BMI GWAS by the Early Growth Genetics Consortium^19^ on 61,111 children aged from 2 to 10 years. For our MR study, we used data from the discovery GWAS meta-analysis involving 39,620 children of European descent (Supplementary Table 1)^19^.

### Mendelian randomization and sensitivity analyses

We performed two-sample MR analyses implemented in the “TwoSampleMR” R package (version 0.5.8)^48^, using the Wald ratio to estimate the effect on BMI for the majority of proteins with a single *cis*-pQTL instrument. To compute the Wald ratios, SNP-exposure effects were used against SNP-outcome effects to compute a MR estimate reflecting the effect (beta) of one standard deviation increase in the level of each protein on standardized BMI values. The GWAS summary statistics for genetic instruments underwent harmonization, aligning them with alleles in the outcome GWAS inferred using allele frequency data. Palindromic variants with an intermediate minor allele frequency (MAF > 0.42) were excluded. The Benjamini-Hochberg method^49^ to compute false discovery rate (FDR) was used to control for multiple testing. MR effects exhibiting an FDR-corrected p-value <0.05 (corresponding to a p-value of < 1.34 x 10^−4^) were considered significant.

### Colocalization analyses

MR estimates might be confounded by linkage disequilibrium (LD), when the SNP-instruments are not causal for the outcome, but instead they are inherited in the same haplotype block (in LD) with a causal SNP. Using colocalization analysis implemented in the coloc R package^50^, we assessed the posterior probability of a genomic region containing a causal variant influencing both the candidate protein level and BMI, examining all SNPs with a minor allele frequency > 0.01 within 1 Mb of the *cis*-pQTLs of the candidate protein. Within the coloc package, we employed default priors of the 'coloc.abf’ function, setting the prior probability of the exposure having a causal variant and the prior probability of the outcome having a causal variant at 1.0x10^−4^, and the prior probability of the exposure and the outcome sharing the same causal variant at 1.0x10^−5^. The results provided posterior probabilities for 4 different scenarios (H0: no association of the genomic locus with either trait; H1: association with BMI but not with the protein; H2: association with the protein but not with BMI; H3: association with BMI and the protein through two different causal SNPs and H4: association with BMI and the protein via one shared causal SNP). A colocalization probability (p4) > 75% was considered robust evidence of colocalization^51^.

### Testing for confounding and horizontal pleiotropy

To test the second and third MR assumption, we investigated potential pleiotropic effects or associations with confounders of the *cis*-pQTL of our candidate proteins, by undertaking phenome-wide association studies (PheWAS) for these *cis*-pQTL in *OpenTargets*^52,53^ (retrieved July 08, 2024). Since BMI accounts for both fat and lean body mass, we undertook a two-step MR^54^ testing potential mediation by adiposity (% fat mass) of the effect of our candidate proteins on BMI. To do this, in the absence of child-specific GWAS on adiposity, we used summary level results from a GWAS for body fat percentage (%BF) in N=155,961 adults of European descent by Hübel et al^23^ (Supplemental Table 1). The two-step MR approach involved a first step, which examined the causal MR relationship between protein levels and %BF, while the second step assessed the MR association between %BF and the outcome, BMI. The indirect MR effect of the proteins on BMI, mediated through %BF, was estimated by calculating the product of the effect of proteins on %BF and the effect of %BF on BMI outcomes. The standard error of this indirect effect and its significance were determined using the Sobel test^55^.

### Replication and reverse MR analyses

To further validate our significant MR associations, we pursued two replication approaches. First, we conducted MR analyses utilizing independent *cis*-genetic instruments identified within the UK Biobank Pharma Proteomics Project (UKB-PPP) study^20^ by Sun et al. This study involved Olink-based measurements of 2,923 unique proteins^20^ in a cohort of 34,557 individuals of European ancestry. Additionally, we replicated MR analyses for the candidate proteins by utilizing a proteomic GWAS meta-analysis by Emilsson et al. (N= 3,200 European adults)^21^. While there was partial participant overlap between this study and the GWAS by Sun et al.^20^, protein measurements in the Emilsson et al. GWAS were conducted using the SomaLogic platform, enabling a significantly larger number (11,000) of protein measurements^56^. In our replication MR studies, we also tested effects of the candidate proteins on adult BMI. To do this, we used data from the Yengo et al GWAS on N= 693,529 European adults^22^ (Supplemental Table 1).

Finally, for proteins prioritized by our main MR analysis, we undertook a reverse MR to identify if BMI affects levels of the candidate circulating protein and not the opposite. For these analyses, we utilized genome-wide significant (GWAS p-value < 5 x 10^−8^) and independent SNPs as instruments for pediatric BMI from the Vogelezang et al^19^ GWAS. Effects of these SNPs were retrieved from the Suhre et al., Folkersen et al., and Ferkingstad et al., proteomic GWAS. MR estimates were computed using the inverse variance weighted (IVW) method and three other pleiotropy-robust methods (MR-Egger, weighted median, and weighted mode)^57–59^.

### MR power analysis

We assessed the power of our main MR study to detect the identified protein effects on BMI based on the variance explained by the protein-increasing alleles of the *cis*-pQTLs, an alpha level of 1.34 x 10^−4^, and the sample size of the pediatric BMI GWAS using the method described by Brion et al.^60^

### Protein-Protein interaction, pathway enrichment and tissue expression analyses

To investigate the functions of the candidate proteins and explore their interactions, we utilized the Gene Multiple Association Network Integration Algorithm (GeneMANIA) tool^61,62^ For each identified protein, we conducted pathway and process enrichment analyses based on their corresponding genes using various gene ontology resources facilitated by Metascape (https://metascape.org/). Additionally, we performed enrichment analysis via FUMA version 1.5.2 (https://fuma.ctglab.nl)^63^. We investigated whether the genes encoding candidate proteins are targets for existing drugs within the *OpenTargets* repository (https://www.opentargets.org). To explore whether the *cis*-pQTL associated with the candidate protein biomarkers for pediatric BMI showed evidence of being expression quantitative trait loci (eQTLs), we generated a gene expression heatmap using Genotype-Tissue Expression (GTEx) v8 through FUMA^64^.

## Supplementary Information

Supplementary Information 1.

Supplementary Information 2.

## Data Availability

All data used in this study are publicly available. The list of GWAS used and full summary statistics available are presented in the supplementary material (Supplementary Table 1).

## Code availability

R codes used to generate the results of the MR and colocalization analyses are available on Zenodo^65^.

## References

[1] Etelson, D., Brand, D. A., Patrick, P. A. & Shirali, A. Childhood obesity: do parents recognize this health risk?. *Obes. Res.* **11**(11), 1362–8 (2003). 14627757 10.1038/oby.2003.184

[2] Dietz, W. H. Health consequences of obesity in youth: childhood predictors of adult disease. *Pediatrics* **101**(3 Pt 2), 518–25 (1998). 12224658

[3] Dehghan, M., Akhtar-Danesh, N. & Merchant, A. T. Childhood obesity, prevalence and prevention. *Nutr J.* **4**(1), 24 (2005). 16138930 10.1186/1475-2891-4-24 PMC1208949

[4] Daniels, S. R. The consequences of childhood overweight and obesity. *Futur. Child.* **16**(1), 47–67 (2006). 10.1353/foc.2006.0004 16532658

[5] French, S. A., Story, M. & Perry, C. L. Self-esteem and obesity in children and adolescents: a literature review. *Obes. Res.* **3**(5), 479–90 (1995). 8521169 10.1002/j.1550-8528.1995.tb00179.x

[6] Richardson, T. G. et al. Use of genetic variation to separate the effects of early and later life adiposity on disease risk: mendelian randomisation study. *BMJ.* **6**(369), m1203 (2020). 10.1136/bmj.m1203 PMC7201936 32376654

[7] Kavarian, P. N., Mosher, T. L. & Abu El Haija, M. Use of glucagon-like-peptide 1 receptor agonist in the treatment of childhood obesity. *Curr Opin Pediatr.* **36**(5), 542–6 (2024). 39254757 10.1097/MOP.0000000000001379

[8] Yazdanpanah, N. et al. Clinically relevant circulating protein biomarkers for type 1 diabetes: evidence from a two-sample mendelian randomization study. *Diabetes Care* **45**(1), 169–77 (2022). 34758976 10.2337/dc21-1049

[9] Niu, L. et al. Plasma proteome variation and its genetic determinants in children and adolescents. *Nat. Genet.* **57**(3), 635–46 (2025). 39972214 10.1038/s41588-025-02089-2 PMC11906355

[10] Skrivankova, V. W. et al. Strengthening the reporting of observational studies in epidemiology using mendelian randomisation (STROBE-MR): explanation and elaboration. *BMJ* **26**(375), n2233 (2021). 10.1136/bmj.n2233 PMC8546498 34702754

[11] Skrivankova, V. W. et al. Strengthening the reporting of observational studies in epidemiology using mendelian randomization: the STROBE-MR statement. *JAMA* **326**(16), 1614–21 (2021). 34698778 10.1001/jama.2021.18236

[12] Sun, B. B. et al. Genomic atlas of the human plasma proteome. *Nature.* **558**(7708), 73–9 (2018). 29875488 10.1038/s41586-018-0175-2 PMC6697541

[13] Pietzner, M. et al. Mapping the proteo-genomic convergence of human diseases. *Science* **374**(6569), eabj1541 (2021). 34648354 10.1126/science.abj1541 PMC9904207

[14] Ferkingstad, E. et al. Large-scale integration of the plasma proteome with genetics and disease. *Nat. Genet.* **53**(12), 1712–21 (2021). 34857953 10.1038/s41588-021-00978-w

[15] Yao, C. et al. Genome-wide mapping of plasma protein QTLs identifies putatively causal genes and pathways for cardiovascular disease. *Nat. Commun.* **9**(1), 3268 (2018). 30111768 10.1038/s41467-018-05512-x PMC6093935

[16] Chong, M. et al. Novel drug targets for ischemic stroke identified through mendelian randomization analysis of the blood proteome. *Circulation* **140**(10), 819–30 (2019). 31208196 10.1161/CIRCULATIONAHA.119.040180

[17] Lu, T. et al. Circulating proteins influencing psychiatric disease: a mendelian randomization study. *Biol. Psychiatr.* **93**(1), 82–91 (2023). 10.1016/j.biopsych.2022.08.015 36280454

[18] Yoshiji, S. et al. Proteome-wide Mendelian randomization implicates nephronectin as an actionable mediator of the effect of obesity on COVID-19 severity. *Nat. Metab.* **5**(2), 248–64 (2023). 36805566 10.1038/s42255-023-00742-w PMC9940690

[19] Vogelezang, S. et al. Novel loci for childhood body mass index and shared heritability with adult cardiometabolic traits. *PLoS Genet.* **16**(10), e1008718 (2020). 33045005 10.1371/journal.pgen.1008718 PMC7581004

[20] Sun, B. B. et al. Plasma proteomic associations with genetics and health in the UK Biobank. *Nature.* **622**(7982), 329–38 (2023). 37794186 10.1038/s41586-023-06592-6 PMC10567551

[21] Emilsson, V. et al. Co-regulatory networks of human serum proteins link genetics to disease. *Science* **361**(6404), 769–73 (2018). 30072576 10.1126/science.aaq1327 PMC6190714

[22] Yengo, L. et al. Meta-analysis of genome-wide association studies for height and body mass index in ∼700000 individuals of European ancestry. *Hum. Mol. Genet.* **27**(20), 3641–9 (2018). 30124842 10.1093/hmg/ddy271 PMC6488973

[23] Hübel, C. et al. Genomics of body fat percentage may contribute to sex bias in anorexia nervosa. *Am. J. Med. Genet. Part B Neuropsychiatr. Genet. Off. Publ. Int. Soc. Psychiatr. Genet.* **180**(6), 428–38 (2019). 10.1002/ajmg.b.32709 PMC6751355 30593698

[24] Seon, K. et al. Endoglin-targeted cancer therapy. *Curr. Drug Deliv.* **8**(1), 135–143 (2011). 21034418 10.2174/156720111793663570 PMC4353483

[25] Possomato-Vieira JS, Khalil RA. Mechanisms of endothelial dysfunction in hypertensive pregnancy and preeclampsia. In advances in pharmacology [Internet] Elsevier [cited 2024 Jun 5] 361–431 Available from: https://linkinghub.elsevier.com/retrieve/pii/S105435891630031X (2016) 10.1016/bs.apha.2016.04.008 PMC4965238 27451103

[26] Bot, P. T. G. et al. Increased expression of the transforming growth factor-β signaling pathway, endoglin, and early growth response-1 in stable plaques. *Stroke.* **40**(2), 439–47 (2009). 19074480 10.1161/STROKEAHA.108.522284

[27] St-Jacques, S., Forte, M., Lye, S. J. & Letarte, M. Localization of endoglin, a transforming growth factor-β binding protein, and of CD44 and integrins in placenta during the first trimester of pregnancy1. *Biol. Reprod.* **51**(3), 405–13 (1994). 7528549 10.1095/biolreprod51.3.405

[28] Wimmer, M. & Weiskirchen, L. Endoglin trafficking/exosomal targeting in liver cells depends on N-glycosylation. *Cells* **8**(9), 997 (2019). 31466384 10.3390/cells8090997 PMC6769735

[29] Lastres, P. et al. Regulated expression on human macrophages of endoglin, an Arg-Gly-Asp-containing surface antigen. *Eur. J. Immunol.* **22**(2), 393–7 (1992). 1537377 10.1002/eji.1830220216

[30] Kajikawa, M. & Higashi, Y. Obesity and endothelial function. *Biomedicines* **10**(7), 1745 (2022). 35885049 10.3390/biomedicines10071745 PMC9313026

[31] Furuhashi, M., Saitoh, S., Shimamoto, K. & Miura, T. Fatty acid-binding protein 4 (FABP4): pathophysiological insights and potent clinical biomarker of metabolic and cardiovascular diseases. *Clin. Med. Insights Cardiol.* **8s3**, CMC.S17067 (2014). 10.4137/CMC.S17067 PMC4315049 25674026

[32] Hotamisligil, G. S. et al. Uncoupling of obesity from insulin resistance through a targeted mutation in aP2, the adipocyte fatty acid binding protein. *Science.* **274**(5291), 1377–9 (1996). 8910278 10.1126/science.274.5291.1377

[33] Steen, K. A., Xu, H. & Bernlohr, D. A. FABP4/aP2 regulates macrophage redox signaling and inflammasome activation via control of UCP2. *Mol. Cell Biol.* **37**(2), e00282-16 (2017). 27795298 10.1128/MCB.00282-16 PMC5214853

[34] Prentice, K. J., Saksi, J. & Hotamisligil, G. S. Adipokine FABP4 integrates energy stores and counterregulatory metabolic responses. *J. Lipid Res.* **60**(4), 734–40 (2019). 30705117 10.1194/jlr.S091793 PMC6446704

[35] Queipo-Ortuño, M. I. et al. FABP4 dynamics in obesity: discrepancies in adipose tissue and liver expression regarding circulating plasma levels. *PloS One.* **7**(11), e48605 (2012). 23139800 10.1371/journal.pone.0048605 PMC3489666

[36] Ogita H, Rikitake Y, Miyoshi J, Takai Y. Cell adhesion molecules nectins and associating proteins: implications for physiology and pathology. Proc Jpn Acad Ser B. 86 (6) 621–9. (2010) 10.2183/pjab.86.621 PMC3081173 20551598

[37] Galibert, L. et al. Nectin-like protein 2 defines a subset of T-cell zone dendritic cells and is a ligand for class-I-restricted T-cell-associated molecule. *J. Biol. Chem.* **280**(23), 21955–64 (2005). 15781451 10.1074/jbc.M502095200

[38] Zhang, S. et al. Competition of cell adhesion and immune recognition: insights into the interaction between CRTAM and Nectin-like 2. *Structure.* **21**(8), 1430–9 (2013). 23871486 10.1016/j.str.2013.06.006

[39] Lawlor, D. A. et al. Mendelian randomization: using genes as instruments for making causal inferences in epidemiology. *Stat. Med.* **27**(8), 1133–63 (2008). 17886233 10.1002/sim.3034

[40] Dong, S. S. et al. Phenome-wide investigation of the causal associations between childhood BMI and adult trait outcomes: a two-sample Mendelian randomization study. *Genome Med.* **13**(1), 48 (2021). 33771188 10.1186/s13073-021-00865-3 PMC8004431

[41] Coorens, T. H. H. et al. The human and non-human primate developmental GTEx projects. *Nature.* **637**(8046), 557–64 (2025). 39815096 10.1038/s41586-024-08244-9 PMC12013525

[42] Khalyfa, A. et al. Fatty-acid binding protein 4 gene variants and childhood obesity: potential implications for insulin sensitivity and CRP levels. *Lipids Health Dis.* **9**(1), 18 (2010). 20156355 10.1186/1476-511X-9-18 PMC2830195

[43] Lehallier, B. et al. Undulating changes in human plasma proteome profiles across the lifespan. *Nat. Med.* **25**(12), 1843–50 (2019). 31806903 10.1038/s41591-019-0673-2 PMC7062043

[44] Ding, Y. et al. Comprehensive human proteome profiles across a 50-year lifespan reveal aging trajectories and signatures. *Cell.* **188**(20), 5763-5784.e26 (2025). 40713952 10.1016/j.cell.2025.06.047

[45] Sainani, K. Interpreting “Null” Results. *PM&R.* **5**(6), 520–3 (2013). 23790820 10.1016/j.pmrj.2013.05.003

[46] Suhre, K. et al. Connecting genetic risk to disease end points through the human blood plasma proteome. *Nat. Commun.* **27**(8), 14357 (2017). 10.1038/ncomms14357 PMC5333359 28240269

[47] Folkersen, L. et al. Genomic and drug target evaluation of 90 cardiovascular proteins in 30,931 individuals. *Nat. Metab.* **2**(10), 1135–48 (2020). 33067605 10.1038/s42255-020-00287-2 PMC7611474

[48] Hemani, G. et al. The MR-Base platform supports systematic causal inference across the human phenome. *elife* **7**, e34408 (2018). 29846171 10.7554/eLife.34408 PMC5976434

[49] Benjamini, Y. & Hochberg, Y. Controlling the false discovery rate: a practical and powerful approach to multiple testing. *J. R. Stat. Soc. Ser. B. Stat. Methodol.* **57**(1), 289–300 (1995).

[50] Giambartolomei, C. et al. Bayesian test for colocalisation between pairs of genetic association studies using summary statistics. *PLoS Genet.* **10**(5), e1004383 (2014). 24830394 10.1371/journal.pgen.1004383 PMC4022491

[51] Foley, C. N. et al. A fast and efficient colocalization algorithm for identifying shared genetic risk factors across multiple traits. *Nat. Commun.* **12**(1), 764 (2021). 33536417 10.1038/s41467-020-20885-8 PMC7858636

[52] Ghoussaini, M. et al. Open targets genetics: systematic identification of trait-associated genes using large-scale genetics and functional genomics. *Nucleic Acids Res.* **49**(D1), D1311-20 (2021). 33045747 10.1093/nar/gkaa840 PMC7778936

[53] Ochoa, D. et al. Open targets platform: supporting systematic drug–target identification and prioritisation. *Nucleic Acids Res.* **49**(D1), D1302-10 (2021). 33196847 10.1093/nar/gkaa1027 PMC7779013

[54] Burgess, S., Daniel, R. M., Butterworth, A. S. & Thompson, S. G. The EPIC-interact consortium. Network mendelian randomization: using genetic variants as instrumental variables to investigate mediation in causal pathways. *Int. J. Epidemiol.* **44**(2), 484–95 (2015). 25150977 10.1093/ije/dyu176 PMC4469795

[55] Sobel Test. In: The SAGE encyclopedia of communication research methods [Internet] 2455 Teller Road Thousand Oaks California 91320: SAGE Publications Inc [cited 2024 Dec 6] Available from: https://methods.sagepub.com/reference/the-sage-encyclopedia-of-communication-research-methods/i13518.xml (2017)

[56] Gold L, Ayers D, Bertino J, Bock C, Bock A, Brody EN, et al. Aptamer-based multiplexed proteomic technology for biomarker discovery. Gelain F (eds). PLoS ONE. 5 (12) e15004. (2010) 10.1371/journal.pone.0015004 PMC3000457 21165148

[57] Bowden, J., Davey Smith, G. & Burgess, S. Mendelian randomization with invalid instruments: effect estimation and bias detection through egger regression. *Int. J. Epidemiol.* **44**(2), 512–25 (2015). 26050253 10.1093/ije/dyv080 PMC4469799

[58] Bowden, J., Davey Smith, G., Haycock, P. C. & Burgess, S. Consistent estimation in mendelian randomization with some invalid instruments using a weighted median estimator. *Genet. Epidemiol.* **40**(4), 304–14 (2016). 27061298 10.1002/gepi.21965 PMC4849733

[59] Hartwig, F. P., Davey Smith, G. & Bowden, J. Robust inference in summary data Mendelian randomization via the zero modal pleiotropy assumption. *Int. J. Epidemiol.* **46**(6), 1985–98 (2017). 29040600 10.1093/ije/dyx102 PMC5837715

[60] Brion, M. J. A., Shakhbazov, K. & Visscher, P. M. Calculating statistical power in Mendelian randomization studies. *Int. J. Epidemiol.* **42**(5), 1497–501 (2013). 24159078 10.1093/ije/dyt179 PMC3807619

[61] Franz, M. et al. GeneMANIA update 2018. *Nucleic Acids Res.* **46**(W1), W60-4 (2018). 29912392 10.1093/nar/gky311 PMC6030815

[62] Mostafavi, S. et al. GeneMANIA: a real-time multiple association network integration algorithm for predicting gene function. *Genome Biol.* **9**(Suppl 1), S4 (2008). 18613948 10.1186/gb-2008-9-s1-s4 PMC2447538

[63] Fitzgerald, J. et al. Thirteen independent genetic loci associated with preserved processing speed in a study of cognitive resilience in 330,097 individuals in the UK biobank. *Genes.* **13**(1), 122 (2022). 35052462 10.3390/genes13010122 PMC8774848

[64] Watanabe, K., Taskesen, E., van Bochoven, A. & Posthuma, D. Functional mapping and annotation of genetic associations with FUMA. *Nat. Commun.* **8**(1), 1826 (2017). 29184056 10.1038/s41467-017-01261-5 PMC5705698

[65] Avocegamou R. Proteome_MR_Analysis [Internet] Zenodo [cited 2025 Mar 8] Available from: https://zenodo.org/doi/10.5281/zenodo.14969499 (2025)

